# Food Insecurity Is Associated with Low Adherence to the Mediterranean Diet and Adverse Health Conditions in Portuguese Adults

**DOI:** 10.3389/fpubh.2018.00038

**Published:** 2018-02-21

**Authors:** Maria João Gregório, Ana M. Rodrigues, Pedro Graça, Rute Dinis de Sousa, Sara S. Dias, Jaime C. Branco, Helena Canhão

**Affiliations:** ^1^EpiDoC Unit, Centro de Estudos de Doenças Crónicas (CEDOC) da NOVA Medical School, Universidade Nova de Lisboa (NMS/UNL), Lisboa, Portugal; ^2^Faculdade de Ciências da Nutrição e Alimentação da Universidade do Porto, Porto, Portugal; ^3^Programa Nacional para a Promoção da Alimentação Saudável, Direção-Geral da Saúde, Lisboa, Portugal; ^4^EpiSaúde Scientific Association, Évora, Portugal; ^5^Sociedade Portuguesa de Reumatologia, Lisboa, Portugal; ^6^Rheumatology Research Unit, Instituto de Medicina Molecular, Lisboa, Portugal; ^7^Unidade de Investigação em Saúde (UI), Escola Superior de Saúde do Instituto Politécnico de Leiria, Leiria, Portugal; ^8^Serviço de Reumatologia do Hospital Egas Moniz – Centro Hospitalar Lisboa Ocidental (CHLO-E.P.E.), Lisboa, Portugal; ^9^Escola Nacional de Saúde Pública da Universidade Nova de Lisboa, Lisboa, Portugal; ^10^Serviço Reumatologia, Centro Hospitalar Lisboa Central-HSM, Lisboa, Portugal

**Keywords:** economic crisis, food insecurity, Mediterranean diet, non-communicable diseases, health-related quality of life, health resources consumption

## Abstract

**Background:**

Food insecurity is a limited or uncertain access to the adequate food and is a significant public health problem. We aimed to assess determinants of food insecurity and the corresponding health impact in Portugal, a southern European country that faced a severe economic crisis.

**Methods:**

Data were derived from the Epidemiology of Chronic Diseases Cohort Study (EpiDoC), a population-based cohort of 10,661 individuals that were representative of the Portuguese adult population and followed since 2011. A cross-sectional analysis of the third wave of evaluation (EpiDoC 3) was performed between 2015 and 2016. Food insecurity was assessed with the household food insecurity psychometric scale. Socioeconomic, demographic, lifestyle, adherence to Mediterranean diet (MD), self-reported non-communicable disease, health-related quality of life (HRQoL) (EQ-5D-3L), physical function (HAQ score), and health resource consumption information was also collected.

**Results:**

The estimated proportion of food insecurity was 19.3% among a total of 5,653 participants. Food insecure households had low adherence to the MD (OR = 0.44; 95% IC 0.31–0.62). In addition, diabetes (OR = 1.69; 95% IC 1.20–2.40), rheumatic disease (OR = 1.67; 95% IC 1.07–2.60), and depression symptoms (OR = 1.50; 95% IC 1.09–2.06) were independently associated with food insecurity. On average, food insecure households had a lower HRQoL (OR = 0.18; 95% IC 0.11–0.31) and a higher disability (OR = 2.59; 95% IC 2.04–3.29). A significantly higher proportion of food insecure households reported being hospitalized (OR = 1.57; 95% IC 1.18–2.07) and had more public hospital medical appointments (OR = 1.48; 95% IC 1.12–1.94) in the previous 12 months.

**Conclusion:**

We found that food insecurity is highly prevalent in Portugal. Food insecurity was associated with low adherence to the MD, non-communicable chronic diseases, lower quality of life, and higher health resource consumption. Therefore, this study provides valuable insight into the relationship between food security and the diet and health of the population during an economic crisis.

## Introduction

Food insecurity is defined as a socioeconomic situation that leads to limited or uncertain access to the nutritious food necessary to maintain a healthy and active life (1). Food insecurity is a significant public health problem and is associated with unhealthy dietary habits and chronic diseases (2–4). Several studies have shown that food insecurity is associated with unhealthy dietary patterns, including reduced intake of fruits, vegetables, and dairy products, and increased intake of energy-dense foods (2, 3). These unhealthy dietary habits are common among food insecure households and may mediate the association between food insecurity and health.

The Mediterranean diet (MD) is a traditional dietary pattern of southern European countries and has been shown to be a healthy diet. This dietary pattern is characterized by a high intake of fruits and vegetables, whole grains, legumes, nuts, and olive oil and a moderate intake of meat. In fact, it is recognized that the MD is associated with lower risk for several chronic diseases, such as cardiovascular events, diabetes, and cancer (5, 6). Some studies have suggested that reduced adherence to the MD in southern European countries during recent years may be related to the European economic crisis (7, 8). This raises questions regarding the impact of the economic crisis on dietary habits, especially those of citizens with food insecurity. Food insecurity may be an important indicator that should be monitored to understand how the socioeconomic situation might be compromising food intake and changing dietary habits.

Food insecurity has been widely studied in the USA and Canada; however, there is a lack of epidemiological data in southern European countries. Addressing food insecurity during an economic crisis is of particular relevance because it is known that periods of economic, political, and social instability tend to notably affect the most vulnerable population strata. In fact, as a result of economic crisis in Portugal, the government adopted austerity policies and large cuts to public expenditure that could lead to inequalities in access to food. Therefore, it is of upmost importance to evaluate food insecurity in a representative sample of the Portuguese population. Moreover, it is important to evaluate the impact of food insecurity on diet and health. The aim of this study was to investigate the prevalence of food insecurity, the association of food insecurity with sociodemographic and economic determinants, and the impact on health status and the consumption of other health resources in a country that faced a recent, severe economic crisis.

## Materials and methods

### Study Design and Participants

**Figure 1:**
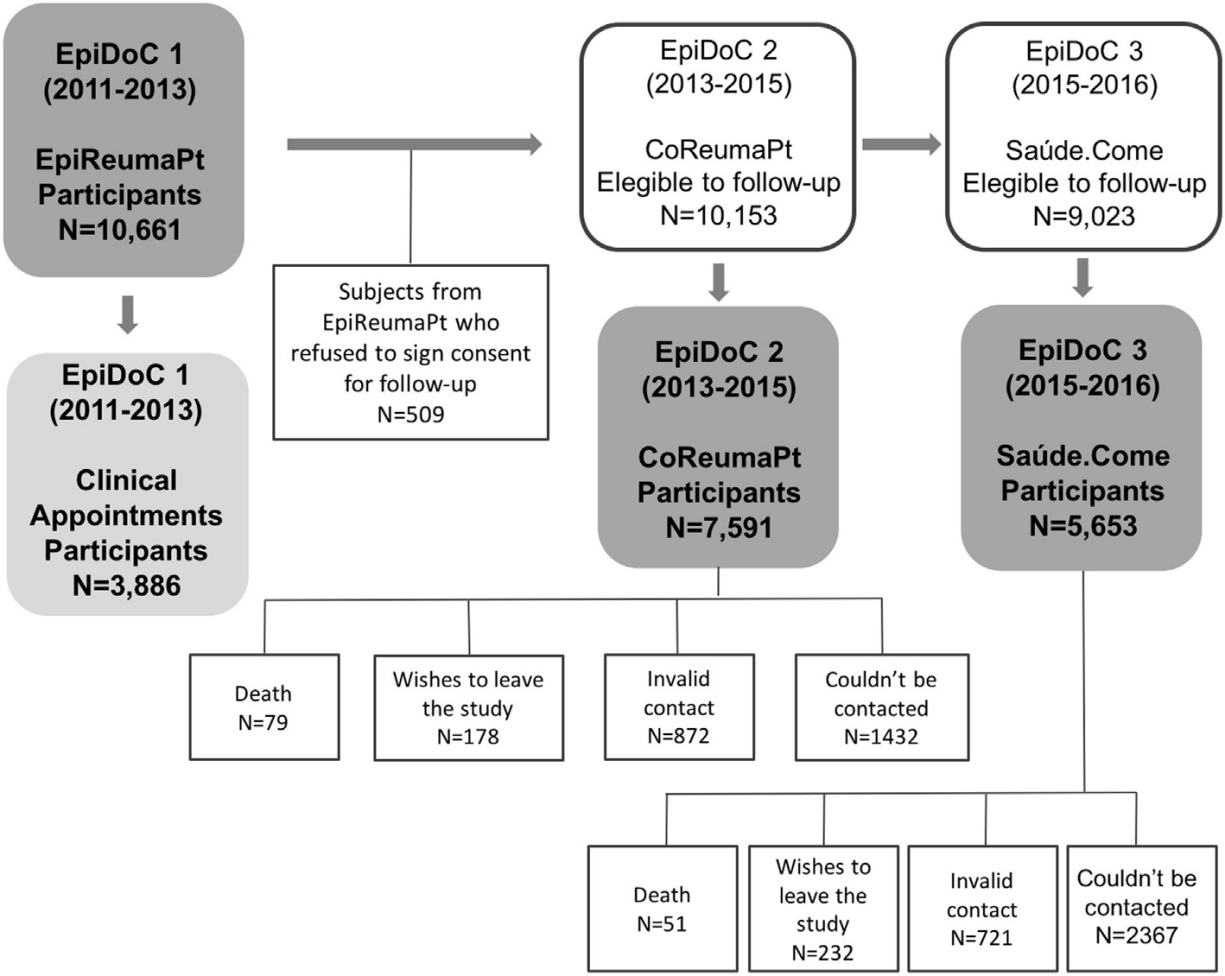
Flowchart of the EpiDoC cohort (consisting of three studies).

The cross-sectional data presented in this study were collected at the third follow-up evaluation wave of the Epidemiology of Chronic Diseases Cohort Study (EpiDoC 3). The EpiDoC cohort was designed to study health determinants and outcomes, chronic non-communicable diseases, and their impact on health resource consumption. The EpiDoC cohort included adults (greater than 18 years old) who were non-institutionalized and living in private households in the mainland and the islands (Azores and Madeira) of Portugal (9). The EpiDoC sample size calculation was performed in order to capture health-related conditions with a prevalence of at least 0.5%, as described elsewhere (10). The estimated prevalence of household food insecurity was notably higher; thus, our sample size had adequate statistical power for the aim of EpiDoC 3. All of the 10,661 participants of the EpiDoC 1 study who signed the informed consent for follow-up and those who provided their telephone number were enrolled in the subsequent follow-up evaluations of the EpiDoC closed cohort studies (EpiDoC 2 and EpiDoC 3) (11). The flowchart of the EpiDoC cohort is described in Figure 1, and EpiDoC 3 included 5,653 participants.

### Data Collection

In the EpiDoC 3 wave of evaluation, a structured questionnaire was applied through computer-assisted phone call interviews by a trained research assistant team from September 1, 2015 to July 28, 2016. Database access is protected by a unique username and password for each research team member. The questionnaire of EpiDoC 3 was constructed using the core questions of the EpiDoC cohort and adding a food insecurity questionnaire and related items. The questionnaire was tested and improved to ensure respondents’ comprehension of the questions and high response rates.

### Measurements

Information on sociodemographics (sex, age, ethnicity, years of education, marital status, and region) were collected in the EpiDoC 1 study. In EpiDoC 3 the structured questionnaire included current household composition, employment status, main source of income, number of children in the household, number of elderly in the household, single-parent family, information regarding the household income perception, and self-reported diseases (high cholesterol level, high blood pressure, diabetes, hyperuricemia, rheumatic disease, allergies, gastrointestinal disease, mental disease, cardiac disease, pulmonary disease, cancer, neurologic disease, and psoriasis).

Health-related quality of life (HRQoL) was assessed using the European Quality of Life questionnaire with five dimensions and three levels (EQ-5D-3L) (12, 13). A higher HRQoL score corresponds to a higher quality of life. Physical function was evaluated based on the Health Assessment Questionnaire (HAQ, 0–3) (14). Higher HAQ scores correspond to reduced functional ability of the individual. We used Portuguese-validated versions of these assessment scales. Data regarding hospitalization in the previous 12 months (Yes/No), number of clinic appointments, doctor visit reduction due to economic difficulties (Yes/No), and stoppage of medication due to economic difficulties (Yes/No) were also recorded. Self-reported height and weight were collected and body mass index (BMI) was calculated and categorized according to the World Health Organization classification (15). Questions concerning lifestyle habits included frequency of alcohol intake (daily, occasionally, never), quantity of alcohol units per week (≤3 alcohol units per week; >3 alcohol units per week but <3 alcohol units per day; >3 alcohol units per day), smoking habits (daily, occasionally, past smoker, never smoked), and frequency and type of physical activity. Physical activity level was classified based on the reported weekly frequency of physical activity: inactive (<1 h/week), moderately active (between 1 and 2.5 h/week), and active (≥2.5 h/week).

To assess adherence to the MD, a PREDIMED (PREvención con DIeta MEDiterránea) questionnaire was applied. This is a 14-item questionnaire with questions based on the number of servings and frequencies of consumption for typical food or food groups of the MD (e.g., olive oil, nuts, fruits, vegetables, pulses, seafood) or questions about the consumption of foods that are not part of the traditional MD (e.g., red or processed meats, sweetened beverages and sweets, commercial bakery, or sugary desserts). A score ≥10 corresponds to a high adherence to the MD, and a score <10 corresponds to a low adherence to the MD (16).

### Case Definition and Assessment

Household food insecurity was assessed using a psychometric scale that was adapted and validated for the Portuguese population from the Brazilian Food Insecurity Scale, which was adapted from the US Household Food Security Survey Module (17). This tool was applied by telephone interview to EpiDoC 3 participants. The household food insecurity scale is a tool applied to the individual and collects data regarding food insecurity status of the entire household. This tool measures both quantitative and qualitative components of food insecurity during the last 3 months. A score ranging from 0 to 14 was obtained from the total number of affirmative responses. According to this score, households were classified into four different categories of food insecurity: food security (if total score was 0), low food insecurity (if total score was between 1 and 5 for households with children and between 1 and 3 for households without children), moderate food insecurity (if total score was between 6 and 9 for households with children and between 4 and 5 for households without children), and severe food insecurity (if total score was between 10 and 14 for households with children and between 6 and 8 for households without children) (18, 19).

### Statistical Analysis

To verify the representativeness of the sample according to the Portuguese population (mainland and islands), we first compared the participants and non-participants of the EpiDoC 3 study with respect to their sociodemographic, socioeconomic, and health status characteristics. Based on this comparison, we adjusted the weights according to stratification by Nomenclature of Territorial Unit for Statistics (NUTS II) region, gender, and age group. Extrapolation weights were computed and used in the subsequent statistical analyses. These were obtained by calibrating the extrapolation weights originally designed for the EpiDoC 1 study sample (9). Absolute frequencies and weighted proportions were used to summarize categorical variables. Continuous variables were described by weighted mean values and SDs. Prevalence estimates for food insecurity were computed as weighted proportions according to region, age groups, gender, employment status, years of schooling, single-parent family, household with children, household with elderly, household income perception, BMI, and adherence to MD. After a descriptive analysis, participants were categorized as “food secure” (high food security) and “food insecure” (including low, moderate, and severe insecurity groups). Subjects with and without food insecurity were compared. Univariable analyses were first performed considering the study design (crude analysis). Multivariate regression models were used to assess dietary, other lifestyles, anthropometric data (BMI), health-related characteristics, and health-care resource consumption differences between individuals with and without food insecurity. In order to adjust the differences between groups, the following potential confounders were included in the logistic regression analysis: age group, gender, educational level, employment status, and NUTS II (adjusted analysis). Significance level was set at 0.05. All analyses were performed using STATA IC version 12 (StataCorp. 2011. Stata Statistical Software: Release 12. College Station, TX, USA: StataCorp LP).

### Ethical Issues and Personal Protection

Details of the ethical issues of the EpiDoC cohort were previously described (9). The EpiDoC 3 study was performed according to the principles established by the Declaration of Helsinki (20) and revised in 2013 in Fortaleza. The study was reviewed and approved by the National Committee for Data Protection and by the NOVA Medical School Ethics Committee (9).

## Results

### Food Insecurity Prevalence and Associated Factors in the Portuguese Adult Population

In EpiDoC 3, a total of 5,653 participants were interviewed. The EpiDoC 3 population did not differ from the Portuguese population (Table 1). Between 2015 and 2016, 19.3% of Portuguese households experienced some level of food insecurity during the previous 3 months. Most food insecure households were in the lower level of food insecurity (14.0%), but 3.5 and 1.8% had moderate and severe food insecurity, respectively.

**Table 1:** Sociodemographic characteristics of the adult Portuguese population: EpiDoC 3 and Census 2011 populations (Portuguese population).

	EpiDoC 3 *n* = 5,653	CENSUS 2011 *n* = 8,657,240
**Gender**		
Female	3,607 (52.49%)	4,585,118 (53.0%)
Age (mean ± SD)	49.64 (18.11)	41.31 (16.28)
**Age group**		
18–29	355 (15.40%)	1,470,782 (17.0%)
30–39	605 (19.08%)	1,598,250 (18.5%)
40–49	1,049 (18.26%)	1,543,392 (17.8%)
50–59	1,143 (15.89%)	1,400,011 (16.2%)
60–69	1,112 (13.72%)	1,186,442 (13.7%)
70–74	491 (6.7%)	496,438 (5.7%)
≥75	893 (10.95%)	961,925 (11.1%)
**Education level**		
>12 years	1,052 (23.99%)	1,741,567 (20.1%)
10–12 years	1,049 (25.60%)	1,560,958 (18.0%)
5–9 years	1,122 (19.55%)	2,134,401 (24.6%)
0–4 years	2,392 (30.86%)	3,239,724 (37.4%)
**NUTS II**		
Norte	1,659 (36.45%)	3,007,823 (34.7%)
Centro	1,087 (23.21%)	1,938,815 (22.4%)
Lisboa	1,131 (24.83%)	2,300,053 (26.6%)
Alentejo	320 (7.22%)	633,691 (7.3%)
Algarve	183 (3.74%)	370,704 (4.3%)
Azores	657 (2.16%)	192,357 (2.2%)
Madeira	611 (2.40%)	213,797 (2.5%)

*Nomenclature of Territorial Units for Statistics (NUTS II) (Norte, Centro, Alentejo, Algarve, Lisboa, Madeira, and Azores)*.

*Sample size is not constant due to missing data*.

*EpiDoC 3—gender (n = 5,648); age (n = 5,648); education (n = 5,648); NUTS II (n = 5,648)*.

Analysis of the socioeconomic factors associated with food insecurity revealed that women (OR = 1.73; 95% IC 1.37–2.18), the less educated (10–12 vs <12 years: OR = 3.10; 95% IC 1.89–5.07; 5–9 vs <12 years: OR = 4.35; 95% IC 2.75–6.90; 0–4 vs <12 years: OR = 7.11; 95% IC 4.49–11.26), individuals aged between 40 and 49 years (OR = 2.28; 95% IC 1.05–4.97), and individuals aged between 50 and 59 years (OR = 2.42; 95% IC 1.10–5.33) had a higher risk of being food insecure. When compared to individuals in a situation of full-time employment, households of individuals who were part-time employed, domestic workers, or unemployed were more likely to be food insecure (OR = 1.68; 95% IC 1.23–2.30). Households living in the Azores (OR = 1.57; 95% IC 1.12–2.20) and Madeira (OR = 1.94; 95% IC 1.32–2.85) also had higher probabilities of being classified as food insecure (Table 2).

**Table 2:** Comparison of sociodemographic, socioeconomic, lifestyles characteristics, and Mediterranean diet (MD) characteristics between households with and without food insecurity.

	Food secure *n* = 4,151	Food insecure *n* = 1,380	Crude OR (95% IC)	*p*-Value	Adjusted OR (95% IC)^a^	*p*-Value
**Gender**						
Female	2,506 (49.82%)	1,033 (63.96%)	1.79 (1.40–2.28)	0.000	1.73 (1.37–2.18)	0.000
**Age group**						
18–29	320 (17.31%)	30 (7.65%)	1		1	
30–39	492 (20.7%)	106 (13.41%)	1.47 (0.60–3.58)	0.401	1.67 (0.73–3.82)	0.244
40–49	782 (18.0%)	256 (19.82%)	2.49 (1.06–5.88)	0.037	2.28 (1.05–4.97)	0.038
50–59	785 (14.28%)	345 (22.95%)	3.64 (1.54–8.60)	0.003	2.42 (1.10–5.33)	0.028
60–69	805 (13.61%)	288 (14.42%)	2.40 (1.01–5.67)	0.046	1.23 (0.56–2.69)	0.613
70–74	347 (6.04%)	128 (8.80%)	3.30 (1.23–8.82)	0.018	1.67 (0.69–4.01)	0.254
≥75	620 (10.07%)	227 (12.94%)	2.91 (1.23–6.88)	0.015	1.30 (0.61–2.81)	0.497
**Education level**						
>12 years	966 (28.04%)	72 (7.56%)	1		1	
10–12 years	862 (27.01%)	174 (20.69%)	2.84 (1.64–4.93)	0.000	3.10 (1.89–5.07)	0.000
5–9 years	795 (18.61%)	307 (23.86%)	4.75 (2.98–7.57)	0.000	4.35 (2.75–6.90)	0.000
0–4 years	1,513 (26.35%)	809 (47.87%)	6.74 (4.33–10.49)	0.000	7.11 (4.49–11.26)	0.000
**NUTS II**						
Norte	1,243 (36.44%)	388 (36.94%)	1		1	
Centro	819 (23.22%)	241 (23.25%)	0.99 (0.74–1.31)	0.934	1.01 (0.75–1.36)	0.958
Lisboa	908 (25.67%)	200 (21.08%)	0.81 (0.57–1.16)	0.249	1.03 (0.67–1.57)	0.891
Alentejo	243 (7.13%)	71 (7.70%)	1.07 (0.73–1.56)	0.745	1.06 (0.72–1.56)	0.772
Algarve	127 (3.53%)	48 (4.24%)	1.19 (0.75–1.87)	0.467	1.27 (0.79–2.02)	0.321
Azores	430 (1.91%)	216 (3.2%)	1.66 (1.23–2.25)	0.001	1.57 (1.12–2.20)	0.009
Madeira	381 (2.10%)	216 (3.57%)	1.68 (1.20–2.35)	0.003	1.94 (1.32–2.85)	0.001
**Single-parent family**	183 (4.58%)	102 (6.84%)	1.24 (1.04–1.48)	0.016	1.16 (0.96–1.40)	0.114
**Employment status**						
Employed full-time	1,870 (53.27%)	360 (32.21%)	1		–	
Employed part-time	134 (4.09%)	54 (3.60%)	1.46 (0.91–2.34)	0.121	–	
Domestic worker	265 (3.95%)	182 (8.14%)	3.41 (2.46–4.72)	0.000	–	
Unemployed	299 (9.43%)	201 (18.74%)	3.28 (1.98–5.45)	0.000	–	
Student	79 (3.63%)	8 (0.95%)	0.43 (0.19–1.01)	0.000	–	
Temporally work disabled	64 (1.19%)	50 (3.33%)	4.63 (2.71–7.89)	0.053	–	
Retired	1,434 (24.44%)	523 (33.04%)	2.24 (1.74–2.88)	0.000	–	
**Employment status**						
Employed full-time/student/temporally work disabled/retired	3,447 (82.52%)	941 (69.53%)	1		1	
Unemployed/employed part-time/domestic worker	698 (17.48%)	437 (30.47%)	2.07 (1.50–2.85)	0.000	1.68 (1.23–2.30)	0.001
**Income perception**						
Living comfortably in the present income	988 (27.41%)	29 (3.29%)	1		1	
Living in the present income	2,015 (48.93%)	253 (19.13%)	3.25 (1.87–5.65)	0.000	2.70 (1.49–4.91)	0.001
Finding difficult in the present income	924 (19.80%)	620 (46.66%)	19.61 (11.32–33.96)	0.000	14.17 (7.75–25.91)	0.000
Finding it very difficult in the present income	200 (3.85%)	471 (30.91%)	66.80 (37.03–120.49)	0.000	45.35 (23.98–85.76)	0.000
**BMI (kg/m^2^)**						
Underweight	71 (2.28%)	17 (1.44%)	1		1	
Normal weight	1,611 (46.23%)	389 (37.06%)	1.27 (0.57–2.84)	0.556	1.30 (0.52–3.23)	0.572
Overweight	1,583 (37.20%)	507 (39.62%)	1.69 (0.77–3.72)	0.191	1.42 (0.57–3.56)	0.448
Obesity	668 (14.29%)	309 (21.88%)	2.43 (1.10–5.40)	0.029	1.73 (0.70–4.31)	0.236
**Alcohol intake**						
Daily	1,259 (30.82%)	299 (24.79%)	0.53 (0.39–0.72)	0.000	0.60 (0.43–0.82)	0.001
Occasionally	1,575 (41.3%)	439 (33.09%)	0.53 (0.40–0.70)	0.000	0.77 (0.56–1.05)	0.101
Never	1,305 (27.88%)	635 (42.12%)	1		1	
**Alcohol intake profile**						
More than 3 alcohol units vs less	151 (4.39%)	34 (3.23%)	–		–	
**Smoking habits**						
Past smoker	900 (21.11%)	243 (21.21%)	1.03 (0.78–1.35)	0.833	1.47 (1.01–2.13)	0.043
Current smoker	594 (19.65%)	206 (21.63%)	1.13 (0.77–1.65)	0.533	1.69 (1.13–2.54)	0.011
Occasionally	59 (1.6%)	15 (0.95%)	0.60 (0.30–1.21)	0.152	1.14 (0.56–2.30)	0.721
Never	2,590 (57.60%)	909 (56.20%)	1		1	
**Physical activity**						
Regular	1,895 (46.67%)	476 (37.16%)	0.28 (0.24–0.32)	0.003	0.85 (0.64–1.13)	0.271
**PREDIMED**						
Low adherence to MD	3,628 (86.9%)	1,299 (94.0%)	1		1	
High adherence to MD	593 (13.1%)	81 (5.9%)	0.42 (0.30–0.58)	0.000	0.44 (0.31–0.62)	0.000
Use olive oil as principal source of fat for cooking	3,720 (89.86%)	1,166 (84.74%)	0.70 (0.53–0.93)	0.015	0.63 (0.46–0.87)	0.005
Olive oil consumption (>4 Tbsp.)	927 (22.33%)	208 (15.07%)	0.55 (0.43–0.70)	0.000	0.60 (0.46–0.78)	0.000
Vegetables consumption (≥2 servings per day)	1,006 (24.24%)	271 (19.64%)	0.80 (0.64–0.99)	0.045	0.77 (0.61–0.97)	0.024
Fruit consumption (≥3 servings per day)	1,450 (34.93%)	323 (23.41%)	0.71 (0.52–0.96)	0.027	0.68 (0.47–1.00)	0.048
Consumption of red meat, hamburger, or sausages (<1 per day)	2,985 (71.91%)	1,130 (81.88%)	2.07 (1.63–2.64)	0.000	1.91 (1.45–2.53)	0.000
Consumption of butter, margarine, or cream (<1 per day)	2,811 (67.72%)	1,017 (73.70%)	1.12 (0.87–1.44)	0.378	1.10 (0.85–1.42)	0.471
Carbonated and/or sugar-sweetened beverages consumption (<1 per day)	3,562 (85.81%)	1,160 (84.06%)	0.94 (0.73–1.22)	0.642	0.80 (0.61–1.06)	0.127
Wine consumption (≥7 cups per week)	1,004 (24.19%)	245 (17.75%)	0.81 (0.62–1.07)	0.144	0.72 (0.54–0.96)	0.027
Pulses consumption (≥3 servings per week)	1,071 (25.80%)	282 (20.43%)	0.72 (0.56–0.93)	0.013	0.76 (0.58–1.01)	0.055
Fish/seafood consumption (≥3 servings per week)	2,494 (60.08%)	613 (44.42%)	0.63 (0.49–0.80)	0.000	0.71 (0.54–0.93)	0.012
Consumption of commercial (not homemade) pastry (<2 servings per week)	2,546 (61.33%)	930 (67.39%)	1.17 (0.93–1.48)	0.189	1.03 (0.78–1.35)	0.849
Nuts consumption (≥3 servings per week)	658 (15.85%)	99 (7.17%)	0.37 (0.27–0.50)	0.000	0.48 (0.34–0.68)	0.000
Preference to eat chicken, turkey, or rabbit instead of beef, pork, hamburgers, or sausages	2,581 (62.57%)	860 (62.45%)	1.22 (0.99–1.51)	0.064	1.22 (0.98–1.52)	0.081
Consumption of boiled vegetables, pasta, rice, or other dishes with a sauce of tomato, garlic, onion, or leeks sautéed in olive oil (≥2 servings per week)	1,825 (43.97%)	526 (38.12%)	0.62 (0.50–0.77)	0.000	0.71 (0.55–0.93)	0.012

*^a^Adjusted for age group, gender, educational level, employment status, and NUTS II*.

*Sample size is not constant due to missing data*.

*EpiDoC 3—gender (n = 5,531); age (n = 5,531); education (n = 5,498); NUTS II (n = 5,531); single-parent family (n = 5,531); employment status (n = 5,523); income perception (n = 5,500); body mass index (BMI) (n = 5,155); alcohol intake (n = 5,503); smoking habits (n = 5,516); physical activity (n = 5,648); PREDIMED (n = 5,531)*.

*Food secure—gender (n = 4,151); age (n = 4,151); education (n = 4,136); NUTS II (n = 4,151); single-parent family (n = 4,151); employment status (n = 4,145); income perception (n = 4,127); BMI (n = 3,933); alcohol intake (n = 4,132); smoking habits (n = 4,143); physical activity (n = 4,151); PREDIMED (n = 4,151)*.

*Food insecure—gender (n = 1,380); age (n = 1,380); education (n = 1,362); NUTS II (n = 1,380); single-parent family (n = 1,380); employment status (n = 1,378); income perception (n = 1,373); BMI (n = 1,222); alcohol intake (n = 1,371); smoking habits (n = 1,373); physical activity (n = 1,380); PREDIMED (n = 1,380)*.

### Participants from Food Insecure Households Had Significantly Lower Adherence to the MD

Adherence to the MD was inversely associated with household food insecurity (OR = 0.44; 95% IC 0.31–0.62), even after adjustment for age, gender, educational level, employment status, and NUTS II. Individuals from food insecure households had a lower tendency to use olive oil as principal source of fat for cooking (OR = 0.63; 95% IC 0.46–0.87) and reduced consumption of vegetables ≥2 servings per day (OR = 0.77; 95% IC 0.61–0.97), fruit ≥3 servings per day (OR = 0.68; 95% IC 0.47–1.00), fish or seafood ≥3 servings per week (OR = 0.71; 95% IC 0.54–0.93), and nuts ≥3 servings per week (OR = 0.48; 95% IC 0.34–0.68) (Table 2).

### Participants from Food Insecure Households Had a Significantly Lower Quality of Life, Physical Functioning, and Consumed More Health-care Resources

To investigate the association of food insecurity with self-reported non-communicable diseases, weighted proportions were adjusted for age, gender, educational level, employment status, and NUTS II. We found that diabetes (OR = 1.69; 95% IC 1.20–2.40), pulmonary disease (OR = 1.67; 95% IC 1.14–2.45), and rheumatic disease (OR = 1.67; 95% IC 1.07–2.60) were independently associated with household food insecurity. The individuals of food insecure households also had a lower quality of life (EQ-5D-3L score: OR = 0.18; 95% IC 0.11–0.31), a higher physical disability (HAQ score: OR = 2.59; 95% IC 2.04–3.29), and a higher risk of having depression symptoms (OR = 1.50; 95% IC 1.09–2.06). Individuals from food insecure households had been more often hospitalized (OR = 1.57; 95% IC 1.18–2.07), had gone more frequently to medical appointments in hospitals (OR = 1.48; 95% IC 1.12–1.94) since last contact, and presented a higher number of medical appointments in the public sector (OR = 1.07; 95% IC 1.04–1.11). Individuals of food insecure households were more likely to have reported stopping medication (OR = 5.13; 95% IC 3.86–6.82) and reducing doctor visits due to economic constraints (OR = 4.23; 95% IC 3.09–5.78) (Table 3).

**Table 3:** Comparison of health status and healthcare resources consumption between households with and without food insecurity.

	Food secure *n* = 4,151	Food insecure *n* = 1,380	Crude OR (95% IC)	*p*-Value	Adjusted OR (95% IC)	*p*-Value
Number of non-communicable diseases (self-reported)	0.76 (±1.04)	1.14 (±1.49)	–	–	–	
**Non-communicable diseases (self-reported)**						
High blood pressure	1,263 (22.47%)	567 (34.07%)	1.78 (1.43–2.23)	0.000	1.23 (0.94–1.62)^a^	0.134
Diabetes	433 (7.53%)	234 (15.18%)	2.20 (1.56–3.10)	0.000	1.69 (1.20–2.40)^a^	0.003
High cholesterol level	1,244 (23.27%)	546 (33.56%)	1.67 (1.33–2.08)	0.000	1.19 (0.93–1.52)^a^	0.166
Hyperuricemia	94 (1.84%)	31 (2.24%)	1.23 (0.75–2.00)	0.417	1.16 (0.71–1.91)^a^	0.553
Pulmonary disease	135 (2.24%)	71 (5.03%)	2.32 (1.59–3.36)	0.000	1.67 (1.14–2.45)^a^	0.008
Cardiac disease	467 (9.13%)	220 (12.64%)	1.44 (1.12–1.87)	0.007	1.24 (0.92–1.68)^a^	0.160
Gastrointestinal disease	369 (8.25%)	168 (11.63%)	1.46 (1.07–2.01)	0.017	1.21 (0.87–1.68)^a^	0.254
Neoplastic disease	219 (4.38%)	93 (5.53%)	1.28 (0.89–1.84)	0.188	1.06 (0.73–1.55)^a^	0.755
**Non-communicable diseases (diagnosed)**						
Diagnosis of rheumatic disease	325 (5.79%)	183 (11.7%)	2.16 (1.51–3.08)	0.000	1.67 (1.07–2.60)^b^	0.010
**Mental health**						
Depression	285 (4.96%)	165 (10.89%)	2.34 (1.77–3.11)	0.000	1.50 (1.09–2.06)^b^	0.012
Anxiety	190 (3.68%)	78 (4.22%)	1.15 (0.80–1.66)	0.437	0.79 (0.52–1.21)^b^	0.285
**Quality of life and physical function**						
EQ-5D-3L score (0–1)	0.83 (0.25)	0.64 (0.36)	0.11 (0.08–0.17)	0.000	0.18 (0.11–0.31)^b^	0.000
HAQ score (0–3)	0.27 (0.46)	0.68 (0.81)	2.99 (2.47–3.62)	0.000	2.59 (2.04–3.29)^b^	0.000
**Healthcare resources consumption**						
Was hospitalized since last contact	498 (9.86%)	223 (15.91%)	1.73 (1.35–2.21)	0.000	1.57 (1.18–2.07)^a^	0.002
Went to medical appointments since last contact	3,796 (90.43%)	1,283 (92.40%)	1.29 (0.91–1.81)	0.148	1.11 (0.75–1.64)^a^	0.601
Went to medical appointments in hospitals since last contact	1,375 (36.17%)	636 (48.62%)	1.67 (1.31–2.13)	0.000	1.48 (1.12–1.94)^a^	0.005
Went to medical appointments in primary health care centers since last contact	2,940 (81.65%)	1,119 (86.225)	1.41 (1.07–1.85)	0.015	1.27 (0.92–1.76)^a^	0.149
Number of medical appointments in public sector since last contact	2.87 (±3.48)	4.80 (±5.87)	1.11 (1.08–1.13)	0.000	1.07 (1.04–1.11)^a^	0.000
Number of medical appointments in private sector since last contact	1.27 (±3.77)	0.99 (±3.77)	0.97 (0.92–1.02)	0.217	1.00 (0.95–1.05)^a^	0.956
Chronic disease management difficulties						
Medication non-adherence due to economic constrains	168 (3.07%)	324 (18.67%)	7.26 (5.53–9.54)	0.000	5.13 (3.86–6.82)^a^	0.000
Reduction in visits to medical appointments due to economic constrains	223 (3.89%)	285 (16.99%)	5.06 (3.86–6.62)	0.000	4.23 (3.09–5.78)^a^	0.000

*^a^Adjusted for age group, gender, educational level, employment status, and NUTS II*.

*^b^Adjusted for age group, gender, educational level, employment status, NUTS II, number of non-communicable, and diagnosis of rheumatic disease*.

*Sample size is not constant due to missing data*.

*Promoting food security—number of non-communicable diseases (n = 5,132); high blood pressure (n = 5,404); diabetes (n = 5,454); high cholesterol level (n = 5,364); pulmonary disease (n = 5,480); cardiac disease (n = 5,451); gastrointestinal disease (n = 5,465); neoplastic disease (n = 45,485); hyperuricemia (n = 5,464); depression symptoms (n = 5,430); anxiety symptoms (n = 5,430); rheumatic disease (n = 5,519); was hospitalized since last contact (n = 5,530); went to medical appointments since last contact (n = 5,079); went to medical appointments in hospitals since last contact (n = 4,869); went to medical appointments in primary health-care centers since last contact (n = 4,874); medication reduction due to economic constrains (n = 5,517); doctor visits reduction due to economic constrains (n = 5,525)*.

*Food secure—number of non-communicable diseases (n = 3,878); high blood pressure (n = 4,053); diabetes (n = 4,096); high cholesterol level (n = 4,027); pulmonary disease (n = 4,112); cardiac disease (n = 4,102); gastrointestinal disease (n = 4,110); neoplastic disease (n = 4,116); hyperuricemia (n = 4,097); depression symptoms (n = 4,070); anxiety symptoms (n = 4,070); rheumatic disease (n = 4,140); was hospitalized since last contact (n = 4,150); went to medical appointments since last contact (n = 3,796); went to medical appointments in hospitals since last contact (n = 3,565); went to medical appointments in primary health-care centers since last contact (n = 3,565); medication reduction due to economic constrains (n = 4,142); doctor visits reduction due to economic constrains (n = 4,147)*.

*Food insecure—number of non-communicable diseases (n = 1,254); high blood pressure (n = 1,351); diabetes (n = 1,358); high cholesterol level (n = 1,337); pulmonary disease (n = 1,368); cardiac disease (n = 1,349); gastrointestinal disease (n = 1,355); neoplastic disease (n = 1,369); hyperuricemia (n = 1,367); depression symptoms (n = 1,360); anxiety symptoms (n = 1,360); rheumatic disease (n = 1,379); was hospitalized since last contact (n = 1,380); went to medical appointments since last contact (n = 1,283); went to medical appointments in hospitals since last contact (n = 1,304); went to medical appointments in primary health-care centers since last contact (n = 1,309); medication reduction due to economic constrains (n = 1,375); doctor visits reduction due to economic constrains (n = 1,378)*.

## Discussion

Using a large population-based database from Portugal, a southern European country that faced a severe economic crisis, we found that the major determinants of food insecurity were unemployment or precarious employment conditions, single-parent family, low education level, and insufficient household income perception. These findings are in accordance with previous studies. The national survey on food insecurity in Canada found that lone-parent families, in particular those headed by women, were the most vulnerable to food insecurity (21). Furthermore, lone-parent families, unemployment or precarious employment condition, low education, and low income are sociodemographic and economic determinants of food insecurity reported worldwide (21, 22). In terms of the employment situation, some studies have highlighted the severe impact of a precarious employment situation on health outcomes, even in comparison with an unemployment situation (23).

The estimated prevalence of food insecurity in Portugal between 2015 and 2016 was 19.3%. The Azores and Madeira Islands were the two regions most affected by food insecurity. A previous study conducted in Portugal in 2003, reported a food insecurity prevalence of 8.1% (24). Between 2005 and 2006, data from the last Portuguese National Health Survey estimated a food insecurity prevalence of 16.7% (25). Comparisons between these studies should be made carefully due to the different methodological approaches in terms of sampling method and food insecurity measurement instrument (24, 25). Nevertheless, our study showed a markedly higher prevalence of food insecurity that cannot be explained solely by methodological differences and might suggest an increasing trend in the prevalence of food insecurity during the last 10 years.

Food insecurity was significantly associated with low adherence to the MD. Lower levels of MD adherence were also found among the lower socioeconomic groups in previous studies (26). Food insecurity has been associated with unhealthy dietary patterns, such as a reduced intake of fruit, vegetables, and dairy products and an increased intake of energy-dense foods among food insecure individuals (2, 3). Our data did not show a significant association between food insecurity and overweight individuals or obesity after adjusting for socioeconomic factors; however, there was a trend toward higher rates of overweight individuals and obesity in food insecure households. These results support findings in other countries where social inequalities have been associated with some determinants of obesity (27–29) and other diet-related non-communicable diseases. Unhealthy dietary habits and lower levels of physical activity were previously identified as determinants of obesity and appear to be more common among the lower socioeconomic groups (30, 31).

We have examined the impact of food insecurity on health and health-related issues and found that subjects with food insecurity reported worse HRQoL and more physical disability when compared to subjects without food insecurity. A higher proportion of subjects with food insecurity were found to have diabetes and rheumatic diseases than those with food security. In fact, our results agree with those from other countries that found strong evidence that vulnerable people, who commonly live in food insecure conditions, have a higher risk of poor health (32, 33). Studies have found that socioeconomically vulnerable groups experience higher mortality and morbidity rates for coronary heart disease (34), atherosclerosis, type 2 diabetes mellitus (35), and some cancers (36). Our study also revealed that a high proportion of subjects with food insecurity reported mental illness in the form of self-reported depression symptoms. The consequences of food insecurity in mental health, namely anxiety and depression symptoms, are well-documented in the literature (37). The causal mechanism is not clear and may be bidirectional. Individuals with food insecurity more often reported difficulties in chronic disease management because they experience more frequent non-adherence to medication and reduced medical appointments due to economic constraints. These difficulties in chronic disease management worsen their health status and decrease disease control. In line with our findings, Berkowitz et al. found that food insecurity is strongly associated with medication underuse (38). The present study also showed that food insecurity was associated with a higher proportion of hospitalizations and clinical appointments, stressing that poor disease control in subjects of food insecurity leads to higher health resource consumption. Tarasuk et al. showed that household food insecurity is a predictor of healthcare utilization and cost in Canada (39).

Food insecurity requires improved collaboration between social protection policies and food and nutrition national policies. Social protection policies, particularly in times of economic crises, are important to ensure adequate socioeconomic conditions for the most vulnerable. Of particular concern for these individuals is the access to healthy food. Food and nutrition national policies should address food insecurity questions in their action plans. This could be accomplished by ensuring that food aid programs are providing healthy food baskets for low-income individuals and empowering these vulnerable individuals for healthy eating. Several countries, especially European countries, fail in their food aid programs due to a low capacity to provide nutritionally adequate foods (40). Toward this end, Portugal has recently developed and implemented a new food aid program for low-income households, which considers the nutritional and food recommendations for healthy eating (41).

Our study has some limitations. Due to the cross-sectional nature of the data, it is not possible to establish causal associations between food insecurity and diet and health outcomes. Food insecurity was assessed based on individual self-perception, and the tool used does not allow us to identify how food insecurity differently affects each member of the household. Several strengths should also be pointed out. Data came from a large, nationally representative sample of the adult population who have been followed since 2011. This study design captured different health and health-related measurements that provided relevant information about determinants and consequences of food insecurity.

In conclusion, this study provided strong evidence that food insecurity is a public health problem that is be associated with a lower adherence to a healthy dietary pattern (MD) and to a higher risk for chronic diseases and poor disease control. Food insecurity leads to more health resource consumption and hospitalization. Data from this study provide valuable information to increase awareness of the food insecurity problem in southern European countries facing an economic crisis. It also provides a strong argument to encourage policymakers to increase the resources allocated to reduce social inequalities and poverty. The implementation of inter-sectoral policies addressing food insecurity is an urgently needed strategy to reduce the burden of diet-related non-communicable diseases.

## Ethics Statement

Details of ethical issues of EpiDoC cohort were described elsewhere [Rodrigues et al. (9)]. EpiDoC 3 study was performed according to the principles established by the Declaration of Helsinki (World Medical Association, 2013) revised in 2013 in Fortaleza. The study was reviewed and approved by the National Committee for Data Protection and by the NOVA Medical School Ethics Committee [Rodrigues et al. (9)].

## Author Contributions

MG and AR contributed equally to this work. HC, AR, and MG designed the study. AR and MG analyzed and interpreted the data and wrote the paper. JB, PG, RS, SD, and HC gave scientific support and revised the manuscript. All authors discussed the results and implications and commented on the manuscript at all stages.

## Conflict of Interest Statement

The authors declare that the research was conducted in the absence of any commercial or financial relationships that could be construed as a potential conflict of interest.
